# The effect of depression on antiretroviral drug non-adherence among women living with HIV in Gondar health facilities, northwest Ethiopia: a prospective cohort study

**DOI:** 10.3389/fpsyt.2025.1488183

**Published:** 2025-04-25

**Authors:** Tadele Amare Zeleke, Tadesse Awoke Ayele, Zewditu Abdissa Denu, Lillian Mwanri, Telake Azale

**Affiliations:** ^1^ Department of Psychiatry, College of Medicine and Health Science, University of Gondar, Gondar, Ethiopia; ^2^ Department of Epidemiology and Biostatics, Institute of Public Health, College of Medicine and Health Sciences, University of Gondar, Gondar, Ethiopia; ^3^ Department of Anesthesia, College of Medicine and Health Sciences, University of Gondar, Gondar, Ethiopia; ^4^ Research Centre for Public Health, Equity and Human Flourishing, Torrens University Australia, Adelaide, SA, Australia; ^5^ Department of Health Promotion and Behavioral Sciences, Institute of Public Health, College of Medicine and Health Sciences, University of Gondar, Gondar, Ethiopia

**Keywords:** depression, ART adherence, women, HIV, Ethiopia

## Abstract

**Background:**

Globally, depression has been recognized as one of the risk factors for poorer outcomes in human immunodeficiency virus (HIV)- affected populations including women living with HIV (WLWHIV). Additionally, depression continues to be a barrier to antiretroviral therapy (ART) adherence. In African countries, including Ethiopia, depression often goes undetected and untreated for extended periods, leading to prolonged health outcomes. Factors such as the lack of awareness about depression and its impact on ART adherence contribute to its poor management. Understanding depression’s role in ART is crucial for generating evidence to improve individuals’ functionality and treatment outcomes. This study aimed to examine the effects of depression on ART non-adherence among WLWHIV in Ethiopia.

**Methods:**

A prospective cohort study was conducted with data collected from 627 study participants who were on stable ART regimens at baseline, 3 months, and 6 months. Depression, the primary exposure variable, was measured using the Patient Health Questionnaire (PHQ-9). Antiretroviral adherence, the dependent variable, was assessed using the Simplified Medication Adherence Questionnaire (SMAQ). Generalized estimating equations (GEEs) were used to examine the association between HIV- related stigma, social support, depression, and ART non-adherence.

**Results:**

The response rates of the study participants in the 2^nd^ and 3^rd^ phases were 99.7% and 94.4%, respectively, with a mean age of 42.27 years (SD ± 10.51). Depressed WLWHIV had a 2.19 times higher incidence of ART non-adherence compared to non-depressed WLWHIV. In panel data analysis, depression, poor social support, and HIV- related stigma were positively associated with ART non-adherence, with adjusted odds ratios of 1.97 [95% confidence interval (CI) (1.35, 2.87)], 2.15 [95% CI (1.05, 4.38)], and 1.56, [95% CI (1.09, 2.25)] respectively.

**Conclusion:**

Depression, poor social support, and HIV- related stigma in women living with HIV were associated with ART non-adherence. Addressing these modifiable barriers could significantly enhance ART adherence in these populations.

## Introduction

Human immunodeficiency virus (HIV) and acquired immunodeficiency syndrome (AIDS) remain major global health challenges. Antiretroviral therapy (ART) is a cornerstone of managing HIV, improving quality of life, and reducing disease progression (1). Adhering to ART is essential for attaining viral suppression, halting disease advancement, and decreasing the spread of HIV (2). However, maintaining adherence can be particularly challenging for women living with HIV (WLWHIV) as they often experience comorbid mental health conditions such as depression (3).

WLWHIV are particularly vulnerable to co-morbidities that affect their mental and physical health and their ability to engage in social activities (4). People living with HIV (PLWHIV) are known to be two to four times more likely to experience depression compared to the general population (5, 6). Moreover, WLWHIV tend to have poorer overall health conditions (7) when compared to both women without HIV and men living with HIV (8).

Following an HIV diagnosis, women often experience stigma, which can lead to social isolation, loneliness, and ultimately depression (9–11). Depression has severe consequences, including increased morbidity, non-adherence to ART, weakened immunity, virological failure, and higher mortality rates (12–16). These consequences undermine effective prevention and treatment efforts for affected women (17, 18) and are associated with faster progression to AIDS and higher mortality rates (19). Despite these significant impacts, depression in WLWHIV remains understudied and undertreated (20).

Antiretroviral therapy for PLWHIV has been shown to increase life expectancy and reduce mortality rate (21–23). However, non-adherence to ART can lead to higher morbidity and mortality. Factors negatively impacting adherence include ART side effects (24–26), lack of support from the healthcare system (27), and the comorbidity of HIV and depression (28, 29). Notably, in low and middle-income countries, depressive symptoms and ART adherence were not significantly influenced by the country’s income (13).

A systematic review and a cross-sectional study in the US among WLWHIV found that depression adversely affected adherence to ART, with depressed women showing poorer adherence than those without depression (30, 31). Similarly, a 12- month retrospective cohort study across eight US states showed that depression significantly reduced adherence to highly active antiretroviral therapy (HAART) and impaired HIV viral control (15). Similarly, in Vietnam, assessments at baseline, 3, 6, and 12 months indicated that depressive symptoms were linked to lower ART adherence (27). Research in Colombia further demonstrated that depression increased the risk of ART non-adherence threefold (32). In Toronto, Ontario, a prospective cohort study conducted from 2007 to 2012 revealed that women with HIV who experienced stress were more likely to have suboptimal ART adherence (33). In Philadelphia, Pennsylvania, a study of 163 participants reported that 63.6% of individuals with depression had poor HAART adherence, compared to 20.1% among those without depression (34). A cross-sectional study in Brazil attributed 46.8% of non-adherence cases to depression (35). A systematic review and meta-analysis in developed countries indicated that depressive symptoms emerged as a significant factor contributing to ART non-adherence, along with the duration of the HIV diagnosis (36). In China, longitudinal data indicated a negative association between depression and ART adherence over time (37). Moreover, a 12-month follow-up study in the United States, with evaluations every 6 months, also confirmed a negative relationship between depression and ART adherence (38).

In Africa, depression may pose a significant barrier to adherence to ART among WLWHIV. A 1-year prospective cohort study conducted in Uganda, which included three assessments throughout the year, found that depression negatively impacted ART adherence among WLWHIV (39). Similarly, a cross-sectional study in Addis Ababa, Ethiopia, was conducted among pregnant women with HIV to determine the rates of non-adherence to treatment. The results showed that 13.5% of non-depressed pregnant women with HIV were non-adherent to their treatment, while 23% of depressed pregnant women with HIV were non-adherent (40).

Having strong social support is a key factor in improving ART adherence (37), even in the presence of depressive symptoms (41). However, depressive symptoms such as loss of interest can hinder women from seeking social support, negatively affecting ART adherence (42). Similarly, studies in Colombia and Pakistan have reported that women living with HIV who had strong social support demonstrated better ART adherence (32, 43).

Goffman defines stigma as an “*attribute that links a person to an undesirable stereotype, leading other people to reduce the bearer from a whole and usual person to a tainted discounted one” (* 44, 45). Depression can be the cause of HIV- related stigma that also affects receiving social support for ART adherence (41, 46). Depression can exacerbate feelings of low self-esteem, which may affect ART medication adherence. There is a bidirectional relationship between depression and HIV- related stigma that can also affect ART adherence (47).

In Africa and India, determinants of ART non-adherence were stigma and poor social support (39, 48, 49). Alcohol could also be a factor for ART non-adherence (33, 48, 50). Gender- based violence (51) and food insecurity are factors related to ART non-adherence for women living with HIV (52). However, in Ethiopia, these factors were not addressed in a longitudinal study.

Despite the availability of effective treatments for mental disorders, over 75% of individuals in low- and middle- income countries do not receive care (53). The mental health needs of WLWHIV are often overlooked due to a lack of research and awareness among healthcare providers and social workers (54, 55). However, early identification and treatment of depression have been shown to improve ART adherence among WLWHIV (56–58). Moreover, findings on the impact of depression on ART adherence remain inconsistent, with some studies suggesting no effect (59), while others indicate a significant negative impact (31). This shows that research on the relationship between depression, social support, HIV- related stigma, and ART non-adherence in Ethiopian WLWHIV is still limited. Therefore, this underscores the need for further research. The mental health needs of WLWHIV are often overlooked in low-income settings such as Ethiopia, and the roles of social support and HIV stigma require more exploration. Cost-effective strategies for screening and managing depression are essential to improve ART adherence and treatment outcomes.

## Materials and methods

### Study design and setting

An institutional- based prospective cohort study was conducted in health facilities in Gondar, northwest Ethiopia. Gondar is located in the northwest of Ethiopia, approximately 728 kilometers away from the capital of the country, Addis Ababa. In Gondar, there are 10 health facilities: one comprehensive specialized hospital, one primary hospital, and eight health centers. Four health facilities, including the University of Gondar Comprehensive Specialized Referral Hospital, Azezo Health Centre, Gondar/Poly Health Centre, and Maraki Health Centre, were selected based on their high patient flow. There were 6,042 adult WLWHIV who had registered for ART follow- up at health facilities in Gondar during the study period. The study was conducted from 1 September 2023 to 30 April 2024.

### Study population

The study population was 1,043 WLWHIV attending the four health facilities with or without depressive symptoms during the study period who were screened at baseline assessment. The participants were included if they had attended for at least 6 months since starting ART treatment and had no history of ART non-adherence. Participants were included regardless of treatment regimens, viral load, CD4 count, and ART side effects. WLWHIV who met the above criteria were randomly selected and enrolled in the study if (a) they were aged 18 years or older, (b) had no plan to move out of the study area before 6 months, and (c) they were well enough to be interviewed, as judged by the interviewers.

### Sample size determination

The sample size was determined using the double population formula, with an exposed-to-non-exposed ratio of 1:1.1. The exposed group included individuals with depression who were ART non-adherent, while the non-exposed group consisted of non-depressed individuals who were also ART non-adherent. A 95% confidence level, 80% power, an odds ratio of 1.9 (40), and a 10% non-response rate were considered in the calculation. As a result, the total sample size for the prospective cohort study was 627, with 299 participants in the exposed group (depressed) and 328 in the non-exposed group (non-depressed).

### Sampling technique

**Figure 1:**
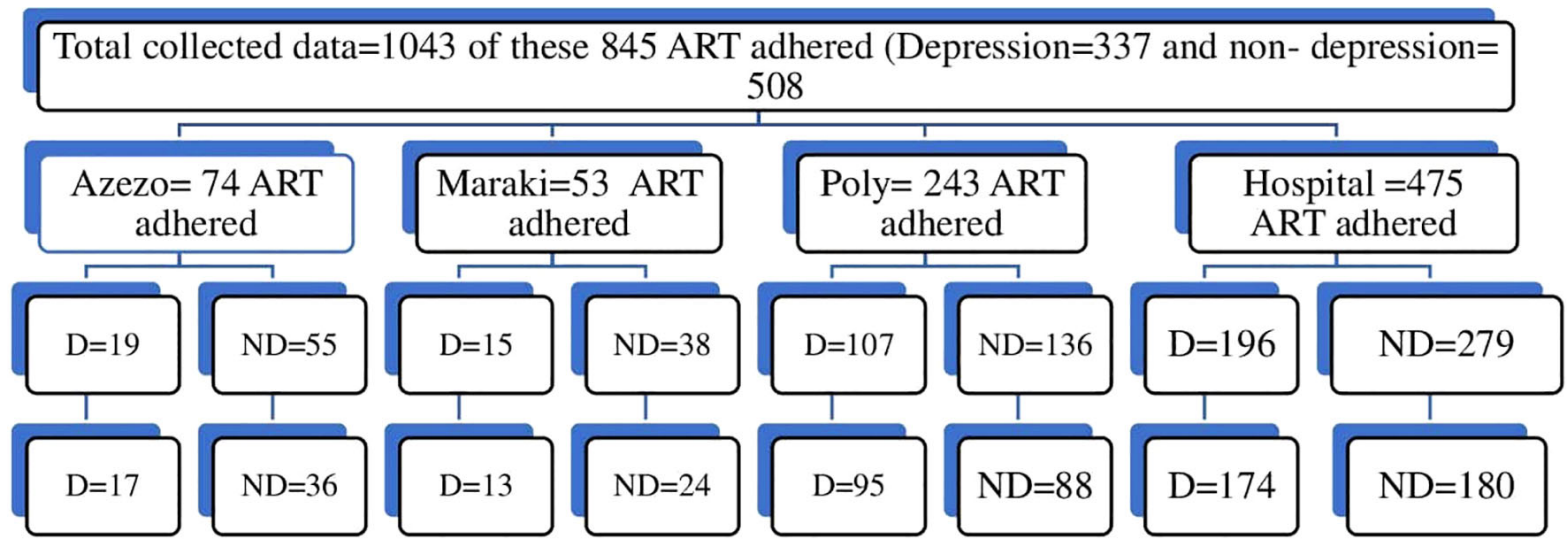
Schematic presentation of the proportional allocation of the sample, 2023/2024. The total sample size for the depression group was 299 out of 337 and for the non-depressed group was 328 out of 508. D, Depression; ND, no depression.

A simple random sampling technique was used to select 299 from depressed and 328 from non-depressed study participants after collecting the baseline data. Reassess depressive symptoms and incidence of ART non-adherence at 3 months and 6 months. The sample size was allocated proportionally to each health facility after identifying the list of potential study subjects from the baseline data for each health facility (**Figure 1**).

### Variables of the study

#### Dependent variable

ART drug non-adherence (yes/no).

#### Independent variables

Sociodemographic factors, including age, educational status, marital status, income, employment status, living arrangement, and residency; clinical factors, including WHO clinical stage of AIDS, ART side effects, types of ART drug, co-morbid medical illness (hypertension, diabetes, tuberculosis, osteomyelitis, cervical cancer, breast cancer, epilepsy, asthma, congested heart failure, kidney disease, etc), family history of mental illness; psychosocial factors, including social support, gender-based violence, HIV status disclosure, and stigma; behavioral factors, including alcohol, cigarettes, and khat.

### Operational definition

ART-non-adherence was defined as answering at least one yes for questions 1, 2, 3, or 5, missing the medication one or more times, or missing medication for 2 or more days, according to the Simplified Medication Adherence Questionnaire (SMAQ) (60).

Depression was defined as scoring 10 and above on the Patient Health Questionnaire (PHQ-9) (61).

Social support was defined by scores on the Oslo Social Support Scale-3 (OSSS-3) (poor was 3 to 8, moderate 9 to 11, and strong 12 to 14) (62).

Current substance use was defined as the use of alcohol, khat, and cigarettes by a WLWHIV in the previous 3 months according to the Alcohol, Smoking, and Substance Involvement Screening Test (ASSIST) (63).

Perceived stigma was assessed by 12 items related to HIV- related stigma, with a higher score indicating increased perceived stigma (64).

Gender- based violence was defined as scoring at least one for the 13 items on the World Health Organization’s Violence Against Women instrument. Physical violence was defined as at least one yes in the six items related to physical violence. Psychological violence was defined as at least one yes in four items related to psychological violence and sexual violence was defined as at least one yes for three items related to sexual violence (65).

Food insecurity was defined according to the Household Food Insecurity Access Scale (HFIAS), with food insecurity increasing as the score increases (66).

### Measurement tools

The SMAQ was used to collect the data. It is a self-administered questionnaire that consists of six items of which four are measured as “yes/no (dichotomous)”, one is Likert-type, and one has two options (60). The reliability and validity of the SMAQ has been studied in different countries (60, 67). The SMAQ was validated in Addis Ababa, Ethiopia among women living with HIV in a two- times measure as the case and control group. At baseline and at the follow-up study, the overall Cronbach’s alpha was 0.72. The concurrent validity of the six items of the SMAQ ranged from moderate to excellent positive correlation (68).

The PHQ-9 was used to assess depression. Participants were asked to rate the frequency of depressive symptoms experienced in the 2 weeks prior to data collection. The total score ranges from 0 to 27. The severity of depression is assessed using a four-point Likert scale: 0 = not at all, 1 = several days, 2 = more than half of the days, and 3 = nearly every day. The score interpretation is as follows: 0–4 points = no depression; 5–9 points = mild depression; 10–14 points = moderate depression; 15–19 points = moderately severe depression; and 20–27 points = severe depression (61). The women who scored 10 and above according to PHQ-9 were referred to the medical and psychiatry unit for further evaluation and counseling for depression.

The Oslo Social Support Scale-3 was used to measure social support for women living with HIV. The level of social support was classified as “poor social support” with a score of 3–8, “moderate social support” with 9–11, and “strong social support” with 12–14. The OSSS-3 consists of three items assessing the number of close intimates, perceived level of concern from others, and perceived ease of getting help from neighbors. The OSSS-3 has good convergent and predictive validity (69).

The risk of harmful substance use was assessed using the modified WHO ASSIST version 3.1 (63), which includes seven items for alcohol use, khat use, and tobacco product use. However, for this study, only the items related to current substance use were considered. This focus on current substance use ensures that the findings are directly relevant to participants’ present behaviors and their immediate impact on ART non-adherence, health outcomes, and psychological wellbeing. The specific items included were: (1) “Have you used any kind of alcohol in the last 3 months?” (Yes/No), (2) “Have you used khat in the last 3 months?” (Yes/No), and (3) “Have you used any tobacco products in the last 3 months?” (Yes/No) (63).

### Data collection procedures

All eligible and consenting women living with HIV were recruited into the cohort. Women living with HIV who had at least 6 months of follow- up (299 with depression and 328 without depression) were randomly recruited from antiretroviral clinics. The data were collected at three time points: at baseline, 3 months, and 6 months. Data collectors (BSc in psychiatry nursing) conducted a face- to- face interview at each health facility with those who were willing to participate in this study and who fulfilled the criteria and were followed from 1 September 2023 to 30 April 2024. Depression, social support, HIV- related stigma, and medication adherence were measured at three time points. However, sociodemographic factors, clinical factors, substance- related factors, intimate partner violence, and food insecurity were collected at baseline.

### Data quality control

Seven trained data collectors and three supervisors participated in the data collection process after 2 days of training. Data collectors had BSc degrees in psychiatry nursing. The supervisor had an MSc in mental health. The training aimed to help the data collectors and supervisors understand the content of the questionnaires, objectives, and ethical issues essential to the study. A pre-test was also conducted on 5% of the sample size at Kola Diba Health Centre to assess the reliability of the questionnaire. The reliability of the SMAQ was 0.71 (Cronbach’s alpha) in the pre-test. The ongoing quality of the data was closely monitored by supervisors and authors of the study every five days in face- to- face meetings and telephone conversations and any problems flagged by the study participants and data collectors were discussed and solved.

### Data processing and analysis

Data were checked for accuracy and completeness, coded and entered into Epi-Data version 3.1, and exported to STATA version 16 for analysis. Descriptive statistics were computed to illustrate the sociodemographic characteristics and clinical, substance, and psychosocial factors of the study participants and to summarize the distribution of the dependent and independent variables. Repeated measurements taken from the same individual over time are often correlated, reflecting within-subject dependencies. Independent variables that had a p-value <0.2 in the bi-variable analysis were exported to the multivariable logistic regression analysis. Thus, in the analysis, correlation within the response has to be accounted for to increase efficiency when estimating regression parameters. To overcome this autocorrelation, a generalized estimating equation (GEE) model was used (70, 71). The GEE method specifies how the average of a response variable of a subject changes with covariates while allowing for the correlation between repeated measurements of the same subject over time. To estimate the best fitting model, quasi-likelihood under the independent model criterion (QIC) for different correlation structures, such as independent, exchangeable, unstructured, and autoregressive, were employed (71, 72). The smallest QIC was found for the autoregressive correlation structure. Thus, it was chosen as the preferred model. The autoregressive correlation structure indicated that two observations taken closer in time within a subject tend to be more highly correlated than two observations taken far apart, which is also supported by theory. The autoregressive correlation structure is advised for use in time series data (73). The final odds ratio (OR) was computed using the “xtgee” command in STATA 16 with the link function (logit) and family (binomial). Time variant variables such as depression, social support, and HIV- related stigma were statistically significant for ART non-adherence. The relative risk ratio (RR) was analyzed to show the incidence of ART non-adherence in the 2^nd^ and 3^rd^ phases.

## Results

### Recruitment flow chart

**Figure 2:**
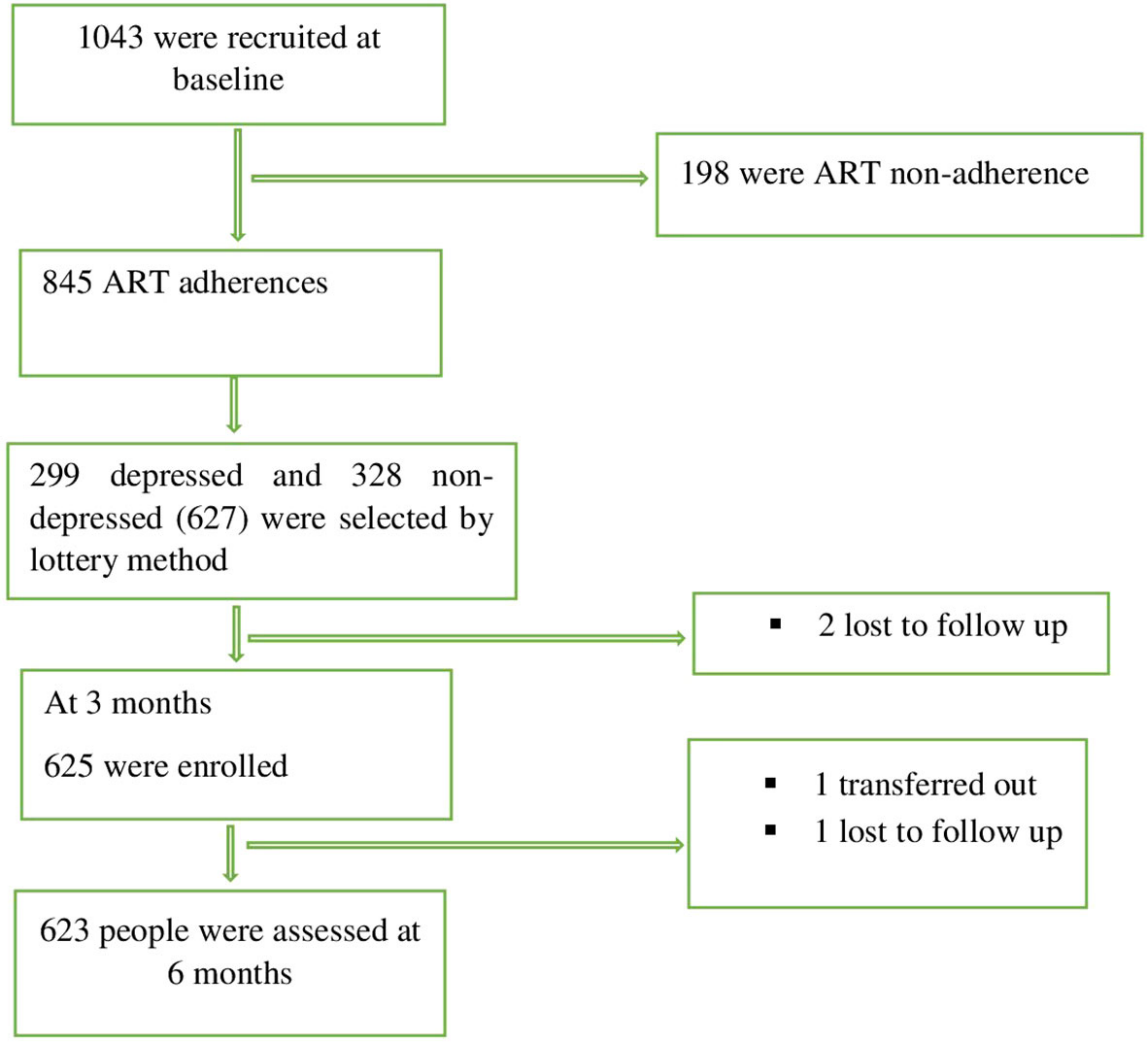
Consort diagram of all participants at recruitment and follow-ups among WLWHIV, 2023-2024.

A flow chart of the recruitment of women living with HIV and the main outcome is presented in **Figure 2**. A total of 627 WLWHIV were eligible to participate; four WLWHIV (0.6%) were excluded during the 2^nd^ and 3^rd^ phases of the follow-up period from the sample due to loss to follow- up (3) or transfer out (1). Finally, 625 WLWHIV in the 2^nd^ phase and 623 in the 3^rd^ phase were included in the analysis, with response rates of 99.7% and 99.4% respectively.

### Socio-demographic characteristics of the participants

Of the total 1,043 potential participants, a total of 627 WLWHIV were enrolled in the cohort study, including 299 with depression and 328 with out depression. Of the enrolled participants, 222 (35.4%) were divorced, the majority were Orthodox Christians (89.8%), and 257 (41.0%) had no formal education. The majority of the study participants (422, 67.3%) had a low-income status, whereas 185(29.5%) were housewives (**Table 1**).

**Table 1:** Sociodemographic characteristics of WLWHIV, 2023–2024 (N=627).

Variable		Frequency	Percent (%)	Depressed vs non-depressed		
				Chi-squared	Fisher’s exact Test	Independent t-test
Age	Mean (42.27) with (SD ± 10.51)					P=0.322
Marital status	Single	53	8.5	p=0.310		
	Married	202	32.2			
	Divorced	222	35.4			
	Widowed	150	23.9			
Religion	Orthodox	563	89.8		P=0.500	
	Muslim	51	8.1			
	Protestant	5	0.4			
	Catholic	6	1.0			
	Seven Day Adventist	2	0.3			
Educational level	No formal education	257	41.0	p=0.431		
	Elementary	168	26.8			
	High school	149	23.8			
	Diploma and above	53	8.5			
Residency	Rural	33	5.3	p=0.792		
	Urban	594	94.7			
Employment status	House wife	185	29.5	p=0.651		
	Unemployed	93	14.8			
	Government employed	76	12.1			
	Private	146	23.3			
	Daily labor	105	16.7			
	Others*	22	3.5			
Income	Low	422	67.3		p=0.253	
	Middle	121	19.3			
	High	84	13.4			
Living arrangement	Alone	84	13.4		p=0.096	
	Family	537	85.6			
	Relatives	6	1.0			

Fisher’s exact test was used for cells with an expected count of less than 5.

The chi-square test was used for categorical variables.

Independent t-test was used for continuous and categorical variables.

Others*= begging, sex worker, waitress.

### Clinical related factors

Among the study participants, 73 (11.6%) were found to have a comorbid medical illness. Additionally, 9 (1.4%) participants reported having a mental illness other than depression (schizophrenia, bipolar disorder, or generalized anxiety disorder). This is because depression was specifically screened in the study and other mental health conditions were also identified and documented based on participant reports and document reviews (**Table 2**).

**Table 2:** Clinically related factors among women living with HIV, 2023–2024 (N=627).

Variable		Frequency	Percentage (%)
Comorbid medical illness	Yes	73	11.6
	No	554	88.4
Mental illness in the family	Yes	18	2.9
	No	609	97.1
Mental illness	Yes	9	1.4
	No	618	98.6
WHO HIV clinical stage	I	598	95.4
	II	18	2.9
	III and IV	11	1.8
ART drug side effect	Yes	6	1.0
	No	621	99.0
ART drug regimens	First line	560	89.3
	Second line	61	9.7
	Third line	6	1
Viral load	Less than 75 copies/mL	600	95.7
	75 copies/mL and more	27	4.3

### Social support, substance use, gender- based violence, and food security characteristics of the study participants

Among the study participants, 349 (55.7%) had moderate social support, while 76 (12.1%) had poor support, with a mean score of 9.1 (SD = 2.2). HIV-related stigma was reported by 286 (45.6%), with a mean score of 24.2 (SD = 6.6).

Regarding substance use, 183 (29.2%) had a history of alcohol use in the previous 3 months, while tobacco use was minimal at 0.3% (2 participants). Gender-based psychological violence was reported by 130 (20.7%) women. Additionally, 213 (34.0%) participants had experienced food insecurity (**Table 3**).

**Table 3:** Social support, substance use, gender- based violence, and food security characteristics of WLWHIV, 2023–2024 (N=627).

Variable		Frequency	Percentage
Social support	Poor	76	12.1
	Moderate	349	55.7
	Strong	202	32.2
HIV- related stigma	Yes	286	45.6
	No	341	54.4
Alcohol use	Yes	183	29.2
	No	444	70.8
Khat chewing	Yes	10	1.6
	No	617	98.4
Tobacco smoking	Yes	2	0.3
	No	625	99.7
Gender- based violence			
Physical	Yes	99	15.8
	No	528	84.2
Psychological	Yes	130	20.7
	No	497	79.3
Sexual	Yes	28	4.5
	No	599	95.5
Disclose HIV status	Yes	450	71.8
	No	177	28.2
Food insecurity	Yes	213	34.0
	No	414	66

### Depression characteristics of the study participants

At baseline, 299 individuals were depressed and ART adherent, while 328 were not depressed and ART adherent. In the second phase (at 3 months), 2 individuals were lost to follow-up, 5 were no longer depressed, and 292 remained depressed. Among the depressed individuals, 39 were ART non-adherent, while among the non-depressed, only 1 was ART non-adherent. By the third phase (at 6 months), of the remaining 278 depressed individuals, 54 were ART non-adherent. Additionally, 17 individuals were no longer depressed, of whom 4 were ART non-adherent.

Among the non-exposed group, at 3 months and 6 months, 16 and 10 WLWHIV had depression, respectively, with no cases of lost follow-up (**Table 4**).

**Table 4:** Characteristics of depression in WLWHIV at 3 and 6 months, 2023–2024.

Exposure and Control groups	First phase		Second phase		Third phase	
	Adherence	Non- adherence	Adherence	Non- adherence	Adherence	Non- adherence
Exposed/depressed						
Depressed	299	0	253 (86.6%)	**39 (13.4%)**	224 (80.6%)	**54 (19.4%)**
Non-depressed	0	0	4 (80.0%)	1 (20.0%)	13 (76.5%)	4 (23.5%)
Non-exposed/non-depressed						
Depressed	0	0	11 (68.8%)	**5 (31.2%)**	6 (60.0%)	**4 (40.0%)**
Non-depressed	328	0	291 (93.3%)	21 (6.7%)	293 (92.1%)	25 (7.9%)

The bold shows ART non-adherence.

### Relative risk ratio of ART non-adherence in the exposed group (depression) and non- depressed WLWHIV

During the second and third phase assessments, measurements were taken among 299 depressed WLWHIV and 328 non-depressed women. Among the total number of depressed women (including both exposed and non-exposed groups), 102 out of 596 (17.1%) had a history of ART non-adherence. In contrast, among the non-depressed WLWHIV (both from exposed and non-exposed groups), 51 out of 652 (7.8%) were ART non-adherent. Depressed WLWHIV were 2.2 times more likely to be ART non- adherent compared to their non-depressed counterparts [RR = 2.19, 95% CI (1.59, 3.00)] (**Table 5**).

**Table 5:** The relative risk of ART non-adherence in depressed and non-depressed WLWHIV at 3 and 6 months, 2023–2024.

		ART non-adherence	ART adherence	95% CI	P-value
Depression	Exposed	102 (17.1%)	494 (82.9%)	2.19 (1.59,3.00)	0.0001
	Non-exposed	51 (7.8%)	601 (92.2%)	Ref	

### Crude odds ratio of ART non-adherence in the 2^nd^ and 3^rd^ phases among women living with HIV

In the third- month measures of depression and ART non-adherence among WLWHIV, the crude odds ratio (COR) of ART non-adherence was 2.23 (95% CI: 1.31,3.83) in depressed WLWHIV compared to non-depressed WLWHIV. In the third phase (at 6 months), the COR of ART non-adherence was 2.66 (95% CI: 1.65, 4.29) in depressed WLWHIV compared to non-depressed WLWHIV (**Table 6**).

**Table 6:** The crude odds ratios (CORs) of ART non-adherence among women living with HIV at 3 and 6 months, 2023–2024.

			ARTNA	ARTA	COR with 95% CI	P-value
In the 2^nd^ phase (at 3 months)	Depression	Yes	44	264	**2.23 (1.31, 3.83)**	**0.003**
		No	22	295	Ref	
In the 3^rd^ phase (at 6 months	Depression	Yes	58	230	**2.66 (1.65, 4.29)**	**0.0001**
		No	29	306	Ref	

ARTNA, ART non-adherence; ARTA, ART adherence. COR which has p-value less than 0.05.

Bold show COR which has p-value less than 0.05.

### Predictors of ART non-adherence among women living with HIV in panel data

In the bi-variable analysis, variables that had a p-value < 0.2 were depression, social support, and HIV- related stigma. Depression, social support, HIV- related stigma, and ART non-adherence were measured three times in a 3-month interval. Depressed women living with HIV were approximately 2 [adjusted odds ratio (AOR)=1.97, 95% CI (1.35, 2.87)] times more likely to be non-adherent than non-depressed WLWHIV. The WLWHIV who had poor social support were approximately 2 [AOR=2.15, 95% CI (1.05, 4.38) times more likely to be ART non- adherent than those who had strong social support. Women living with HIV who experienced HIV- related stigma were 1.56 times [AOR=1.56, 95% CI (1.09, 2.25)] more likely to be non-adherent than their counterparts (**Table 7**).

**Table 7:** Odds ratios from the generalized estimating equation (GEE) model predicting ART non-adherence among WLWHIV (1,875 observations from n=627).

Variable		ART non-adherence		COR with 95% CI	AOR with 95% CI	P-value
		Yes	No			
Depression	Yes	102	792	2.35 (1.66, 3.33)	**1.97 (1.35, 2.87)**	0.0001
	No	51	930	Ref		
Social support	Poor	85	684	2.84 (1.41, 5.75)	**2.15 (1.05, 4.38)**	0.035
	Moderate	59	832	1.62 (0.79, 3.33)	1.53 (0.75, 3.14)	0.246
	Strong	9	206	Ref		
HIV- related stigma	Yes	100	829	2.03 (1.44, 2.87)	**1.56 (1.09, 2.25)**	0.016
	No	53	893	Ref		

AOR factors were significant for ART non-adherence at p<0.05.

Bold indicates in AOR factors were significant for ART non-adherence at p<0.05.

## Discussion

This study examined the longitudinal relationship between depression, social support, HIV-related stigma, and ART non-adherence. The findings indicate that depression remained a consistent and significant predictor of ART non-adherence over time. Additionally, poor social support and HIV-related stigma were both positively associated with ART non-adherence, suggesting that psychosocial factors play a crucial role in influencing treatment adherence among WLWHIV.

### Depression and ART adherence

In the current study, WLWHIV with depression were nearly twice as likely to be ART non- adherent compared to those without depression. This finding is supported by previous research, including a systematic review that reported higher non-adherence to ART among depressed WLWHIV (31), and studies have demonstrated that depression significantly worsens HAART adherence, thereby impacting HIV viral load control (15, 27). One study even found that depression tripled the risk of ART non-adherence (32). Additional research has identified depression as a key factor in suboptimal adherence (33, 74, 75) and a significant barrier to ART adherence (39), with depressed women being at a twofold higher risk of decreased ART uptake and increased treatment interruption compared to men (31). Possible reasons may include depressive symptoms, such as lack of concentration, and sleeping problems could result in forgetfulness, decreased energy, and negative thoughts about self and the effect of ART medication, which in turn could impact missing medical appointments and taking medication on time (76). Moreover, impaired memory, executive dysfunction, engagement in risk behaviors such as alcohol consumption (46), and feelings of hopelessness and apathy, which reduce motivation for self-care, further exacerbate ART non-adherence in this population (42, 77, 78).

### Social support and ART adherence

Women living with HIV with poor social support were approximately twice as likely to be ART non- adherent compared to those with strong social support. This finding aligns with previous research indicating that robust social support enhances adherence, as evidenced by a study from Colombia (32), while another study revealed that low perceived social support is linked to suboptimal ART adherence (16). Moreover, evidence suggests that strong family cohesion and effective caregiver support contribute to improved ART adherence (79). Furthermore, poor social support has been associated with ART non-adherence in several studies (39, 48, 49). Additionally, inadequate social support can lead to challenges such as food insecurity, further compromising ART adherence (80). The underlying mechanism may be a lack of medication reminders and insufficient assistance, including transportation to the clinic and financial support for medication, which ultimately impact ART adherence (76, 80).

### HIV stigma and ART adherence

HIV is widely recognized as a highly stigmatized condition (45, 81, 82), and our study found that HIV- related stigma was positively associated with ART non-adherence. This observation is in line with previous research showing that internalized stigma can predict suboptimal ART adherence (16) and that HIV- related stigma is a significant factor in ART non-adherence (39, 48, 49). One possible explanation is that stigma serves as a major source of stress, leading to depression and anxiety, which in turn undermine the ability of WLWHIV to adhere to their ART regimen (46). Additionally, the negative self-perceptions and shame engendered by stigma may reduce motivation for maintaining treatment (83). Stigma experienced in both healthcare settings and the community can result in social isolation and decreased social support, critical elements for ART adherence. Moreover, to avoid discrimination, women may conceal their HIV status, leading to missed medical appointments and/or avoidance of taking medication in the presence of others (84). Discrimination and stigma impact health through complex pathways, including the induction of stress, internalization of negative stereotypes, physical assault, engagement in harmful coping behaviors such as substance abuse, and unequal access to essential health resources (85, 86).

### Limitation and strength

By utilizing a longitudinal study, this study demonstrates the direct association between depression and ART non-adherence among women living with HIV. This study provides a new perspective to understand the relationships between depression, social support, HIV- related stigma, and ART non-adherence. The limitations of this study were as follows. Depressed women living with HIV were referred to the psychiatry clinic and medical unit, which may have affected ART adherence, and since depression was assessed by a screening tool and self-reporting rather than diagnostic interviews, it was difficult to make accurate depression diagnoses. The other limitation was that data on perceived HIV-related stigma, social support, and ART non-adherence were collected through self-report questionnaires, which may be influenced by social desirability bias. Participants may have provided responses they believed would be viewed positively by others, rather than reflecting their true thoughts or behaviors.

### Conclusion and recommendation

Depression appears to be the biggest risk factor for ART non-adherence in WLWHIV. Depression, poor social support, and HIV- related stigma are significant factors in ART non- adherence among WLWHIV. These findings suggest that interventions targeting depression, strengthening social support, and counseling for HIV- related stigma may be effective in improving ART adherence among women living with HIV. Therefore, a holistic and integrated approach is crucial for improving ART medication adherence. Health care providers have to be given the support needed to provide depression care and counseling for HIV- related stigma and strengthen social support for WLWHIV.

### Implications and future directions

This study highlights the complex relationships between depression, social support, and stigma influencing ART adherence among WLWHIV in Gondar. It emphasizes the need for public health initiatives to address these factors, which are significant barriers to ART adherence. Policymakers, healthcare providers, and social service providers must prioritize these issues. Future research should focus on interventions to reduce depression and stigma while strengthening social support to improve ART adherence in Ethiopia and similar settings.

## Data Availability

The original contributions presented in the study are included in the article/supplementary material. Further inquiries can be directed to the corresponding author.
